# Anti-Műllerian hormone – a prognostic marker for metformin therapy efficiency in the treatment of women with infertility and polycystic ovary syndrome

**Published:** 2012-12-25

**Authors:** M Neagu, C Cristescu

**Affiliations:** “Prof. Dr. P. Sîrbu" Clinical Hospital of Obstetrics and Gynecology, Bucharest, Romania

**Keywords:** AMH, PCOS, metformin

## Abstract

**Background:** The anti- Műllerian hormone (AMH) is secreted in women exclusively by the granulosa cells of the ovarian follicles. The serum level of AMH is a precise marker of follicle pool size. In recent clinical studies of polycystic ovary syndrome (PCOS), the serum levels of AMH were elevated about two to threefold. The use of metformin in women with infertility and PCOS has proved to be efficient: restoring ovulation and reducing metabolic dysfunctions. The aim of our study is to assess AMH as a prognostic marker for metformin therapy efficiency in the treatment of women with infertility and polycystic ovary syndrome (PCOS).

** Methods:** Eleven patients with infertility and PCOS were enrolled; PCOS was diagnosed according to the criteria of Androgen Excess and Polycystic Ovarian Syndrome Society 2006 (AE/PCOS). All patients have received metformin therapy. Serum AMH was recorded before and after 2 months of treatment; the normal laboratory values were 2.0-6.8 ng/ml.

** Results: **The primary serum AMH level of all women in study was very high: 8.99±0.99 ng/ml. After 2 months of treatment with metformin ovulation was restored in all the patients and the serum AMH levels were significantly decreased.

**Conclusions:** In clinical practice, serum AMH levels of women with infertility and PCOS receiving metformin are a useful predictive marker for the treatment efficiency.

## Background

Anti-Műllerian hormone (AMH) is a member of the transforming growth factor ß family (TGF-ß), together with inhibins, activins and other proteins with varied functional roles. Although it has mainly autocrine and paracrine role, the AMH is founded in significant quantity in serum. It is also called “műllerian inhibitor substance" and it was studied for its role in the differentiation of male sexual characteristics during embryonic life. AMH is produced by Sertoli cells of the embryos testis at 7 weeks of gestational age and induce the műllerian ducts regression. In the female embryo, the AMH secretion is absent, allowing development of fallopian tube, uterus and upper part of the vagina from the Műller ducts.

In women, AMH is produced exclusively by the granulosa cells of the ovarian follicles and its secretion begins at puberty and lasts until menopause. Thus, there are low levels of AMH in primordial follicles, maximum expression in the greater preantral and small antral follicles, and again lesser secretion in the final stage of follicular maturation.

AMH has a paracrine role in the ovary, independent from the negative feedback of the gonadotropins. AMH has two roles in follicular development:

- Inhibits the primordial follicles growth; decreasing the recruitment rate, AMH helps prevent early extinguish of the follicular pool.

- Decrease the follicle-stimulating hormone (FSH) responsiveness of preantral and small antral follicles suppressing the FSH-depending aromatase and, also, diminish the luteinizing hormone (LH) receptors, thus helping the selection of the dominant follicle.

AMH serum levels reflect the ovarian follicular reservoir, the number of small antral follicles being correlated with that. The AMH screening can reflect better the ovarian pool size given its stability during the entire cycle, unlike other parameters used, such as inhibin B, estradiol and FSH. In addition, AMH is the first marker that changes before the loss of the follicular pool.

Serum AMH levels are the main marker of the ovarian follicular reservoir used in human assisted reproduction in order to evaluate the ovarian stimulation protocols, its dosage being a part of infertility assessment investigation protocol of the patients in this programs [**10**].

PCOS is the main cause of anovulatory infertility. That is not a specific endocrine pathology with a solitary cause, but a syndrome with different signs and symptoms, none of them being specific. The PCOS incidence in female population of reproductive age is variable according to different authors, between 7-15%, because there is no specific diagnostic test universally accepted [**1,3**].

PCOS is the main common and obvious condition related to chronic anovulation; the pathophysiology of anovulation involves many mechanisms that are acting at each level of the reproductive chain. The statement that PCOS is the common cause of anovulation is inexact, because PCOS is rather a consequence of chronic anovulation. Thus, PCOS can be described more accurate as chronic anovulation with polychistic ovaries [**2**].

In all the patients with PCOS were described levels of seric AMH two to three times higher than normal, but there is no evidence that AMH excess is secondary to the large number of preantral and small antral follicles determined by chronic anovulation or if this excess represents the reason of growth-impairment of this follicles due to a higher intrinsic secretion of the granulosa cells that lead to chronic anovulation.

Ovarian stimulation in female with infertility and PCOS includes the insulin sensitizing agents – biguanides and tiazolidindiones; the most used therapy is represented by metformin [**10**]. Metformin’s path of action is commonly unknown, but is seems that it suppress the hepatic gluconeogenesis. Also, metformin improves the peripheral resistance to insulin, increase the consumption of glucose in skeletal muscles and decrease the intestinal glucose absorption. Metformin enhance insulin action at cell levels by enhancing the caption of glucose in adipose and muscular cells and by increasing the ligation to the insulin receptors [**7**]. 

The beneficial role of metformin on the serum androgens is due to:

- increase the hepatic production of SHBG, thus lowering the circulating free testosterone

- decrease the adrenal androgen production 

- decrease androgen production in ovary

There are a series of metformin treatment protocols, the total daily dose being 1500-2550 mg. The main side effects of metformin are nausea and diarrhea. These side effects can be prevented if metformin is taking during meals. Metformin has been demonstrated to induce regular menstrual cycles, increase ovulation, ameliorate hirsutism and produce a slight weight lose [**8**]. The metformin effects on endocrine parameters are: decrease the free testosterone levels, decrease SHBG, normalization of LH and a slightly increase of FSH levels, insulin decrease, estrone decrease [**9**].

## Methods

Eleven patients with infertility and POCS were enrolled. The PCOS diagnosis was made according to AE/PCOS 2006 criteria: hyperandrogenism (clinical and/or biological), ovarian dysfunction (oligo anovulation and/or polycystic ovary), with exclusion of other causes of hyperandrogenism and polycystic ovary [**4**].

The PCOS diagnosis was apparent during the clinical history; many obvious features for PCOS were found at the majority of patients in the study lot:

- first menstruation after age of 14 in all patients, in 54,54% even after age of 15

- oligo- and/or spaniomenorrhea in all patients

- long term infertility

- first degree relatives in 90,90% of patients had oligomenorrhea and infertility, diabetes mellitus, hypertension, dyslipidemia and obesity.

The general clinic examination signs suggestive for PCOS were isolated and that is why the clinical exam was of little importance in the diagnostic set up. Thus, we have noticed clinical signs of hyperandrogenism in 45,45% of patients such as mild hirsutism, BMI > 25 in 9,09% of patients, and in just 0,09% of cases we found the classical syndrome described by Stein and Leventhal.

The hormonal assessment of patients confirmed the clinical diagnosis of PCOS, detecting hyperandrogenaemia and anovulation in all the patients, also permitting to negate other reason for those. The hormonal assessment consisted of: FSH, LH, free testosterone, AMH, thyroid hormones, prolactin and insulin.

The imagistic evaluation consists of gynecologic transvaginal ultrasonography and hysterosalpingography, these being mandatory in the infertility assessment protocol. No tubal permeability anomalies were detected. Polycystic ovaries were confirmed by ultrasound in all patients. 

Metformin treatment was used in patients with infertility and PCOS for ovarian stimulation in order to obtain ovulatory cycles and, subsequent, gestation; no other ovarian stimulation drugs or ovarian induction therapy was used, also no ovarian drilling was performed [**5,6**].

The daily metformin dosage was 2550 mg split in 3 equal quantities of 850 mg. The treatment started with 850 mg/day during meals for one week, then 850 mg/12 hours for another week, and then 850 mg/8 hours until a gestation was obtained. Metformin administration was continued until the 8th week of gestation. None of the patients has abandoned the treatment [**11**]. 

Side effects of metformin were recorded in 18,18% of the patients, and had consisted of mild nausea and diarrhea, but that didn’t required aborting the treatment or lowering the metformin dose. 

## Results

 After 2 months from the start of the treatment, regulate ovulatory cycles at 28-30 days were obtained in all patients. The restitution of ovulation was confirmed by ultrasonography and by hormonal measurement in day 14 and day 21 of menstrual cycle. The monitoring of ovulation was continued by every patient using fast ovulation tests and/or basal body temperature chart; with regulate sexual contacts mainly in the fertile period.

 Serum AMH levels, measured at all 11 patient in our lot before starting metformin therapy, were considerably high: 8,99±0,99 ng/ml, according to expectation in PCOS patients. Also, the free testosterone and LH levels were elevated, these being evocative for hyperandrogenism and chronic anovulation.

 Serum AMH level after 2 month of metformin treatment were considerable lower and paired the normal laboratory criteria, and were 6,28±0,46 ng/ml. Also, the free testosterone and LH values were normal.

## Conclusions

Serum AMH was recently proposed as diagnostic marker of POCS, due to two pathognomonic fundaments for this syndrome: the large pool of preantral and small antral follicles blocked in this stage and chronic anovulation.

 This study, even with a small number of enrolled patients and with a uniform PCOS study lot, demonstrates the utility of AMH as a prognostic factor of metformin therapy in clinical practice.
